# Attenuation of reactive gliosis in stroke-injured mouse brain does not affect neurogenesis from grafted human iPSC-derived neural progenitors

**DOI:** 10.1371/journal.pone.0192118

**Published:** 2018-02-05

**Authors:** Cecilia Laterza, Naomi Uoshima, Daniel Tornero, Ulrika Wilhelmsson, Anna Stokowska, Ruimin Ge, Milos Pekny, Olle Lindvall, Zaal Kokaia

**Affiliations:** 1 Department of Clinical Sciences, Laboratory of Stem Cells & Restorative Neurology, Lund Stem Cell Center, University Hospital, Lund, Sweden; 2 Department of Anesthesiology, Tokyo Medical University, Nishishinjuku, Shinjuku-ku, Tokyo, Japan; 3 Department of Clinical Neuroscience, Institute of Neuroscience and Physiology, Laboratory of Astrocyte Biology and CNS Regeneration, Center for Brain Repair, Sahlgrenska Academy at the University of Gothenburg, Gothenburg, Sweden; 4 Department of Clinical Neuroscience, Institute of Neuroscience and Physiology, Laboratory of Regenerative Neuroimmunology, Center for Brain Repair, Sahlgrenska Academy at the University of Gothenburg, Gothenburg, Sweden; University of South Florida, UNITED STATES

## Abstract

Induced pluripotent stem cells (iPSCs) or their progeny, derived from human somatic cells, can give rise to functional improvements after intracerebral transplantation in animal models of stroke. Previous studies have indicated that reactive gliosis, which is associated with stroke, inhibits neurogenesis from both endogenous and grafted neural stem/progenitor cells (NSPCs) of rodent origin. Here we have assessed whether reactive astrocytes affect the fate of human iPSC-derived NSPCs transplanted into stroke-injured brain. Mice with genetically attenuated reactive gliosis (deficient for GFAP and vimentin) were subjected to cortical stroke and cells were implanted adjacent to the ischemic lesion one week later. At 8 weeks after transplantation, immunohistochemical analysis showed that attenuated reactive gliosis did not affect neurogenesis or commitment towards glial lineage of the grafted NSPCs. Our findings, obtained in a human-to-mouse xenograft experiment, provide evidence that the reactive gliosis in stroke-injured brain does not affect the formation of new neurons from intracortically grafted human iPSC-derived NSPCs. However, for a potential clinical translation of these cells in stroke, it will be important to clarify whether the lack of effect of reactive gliosis on neurogenesis is observed also in a human-to-human experimental setting.

## Introduction

Ischemic stroke is a leading cause of brain damage, long-term disability and death in humans [1]. Apart from thrombectomy and thrombolysis during the first hours after the insult, which can be applied only to a minority of patients, there are no effective treatments to improve functional recovery in the post-ischemic phase. Over recent years, stem cell-based approaches have emerged as promising experimental tools with a potential for the restoration of brain function also in stroke patients [2]. From a clinical perspective, reprogramming of somatic cells seems attractive for the generation of cells suitable for transplantation in stroke, in particular because this strategy avoids the ethical issues associated with the use of human embryonic stem cells. A bulk of experimental studies has demonstrated that grafted reprogrammed cells can induce functional improvements in stroke models (for references see, e.g., [3]). For example, we have shown that human induced pluripotent stem cell (iPSC)-derived neural stem/progenitor cells (NSPCs), transplanted into mouse and rat models of stroke, improve sensorimotor deficits, differentiate to mature neurons [4, 5], and integrate anatomically and functionally into host neuronal circuitry [6].

For the clinical translation and optimization of their therapeutic efficacy, it is important to understand how the tissue environment in the stroke-injured brain affects the behavior and fate of the grafted cells. One prominent pathological feature of ischemic stroke is reactive gliosis and glial scar formation [7–11]. After stroke, astrocytes undergo prominent changes in morphology, function and expression profile [12–14]. These reactive astrocytes are characterized by cellular hypertrophy and upregulation of glial fibrillary acidic protein (GFAP), which is the main component of the cytoplasmic intermediate filament (IF) system (known also as the nanofilament system) of astrocytes, together with vimentin, nestin and synemin [15–19]. Besides a pivotal role in astrocyte structure, IFs are central players in transducing biomechanical and molecular signals and in regulating astrocyte functions [15, 19]. In *GFAP*^*-/-*^*Vim*^*-/-*^ mice, reactive astrocytes show abundance and distribution comparable to that of wild-type (WT) mice [20], but are not hypertrophic [17, 20] and generate less dense glial scar [21, 22].

Reactive astrocytes have been reported to have a beneficial protective role after brain ischemia [23, 24]. *GFAP*^*-/-*^*Vim*^*-/-*^ mice with attenuated reactive gliosis show increased loss of brain tissue after ischemic stroke induced by middle cerebral artery transection [23]. Reactive astrocytes induced by the ischemic insult assist in repairing the blood–brain barrier, controlling the osmoregulation, counteracting the development of brain edema, limiting immune cell influx, minimizing neuronal death and sealing the lesioned area from the rest of the CNS, thereby limiting the spread of the damage [19, 23, 25–29].

However, reactive astrocytes can also negatively effect the regenerative capacity, for example after neurotrauma [19, 27, 28, 30]. Several reports indicate that reactive gliosis inhibits survival and differentiation of neural progenitor cells *in vitro* and neurogenesis *in vivo* as well as CNS regeneration after injury [20, 31–36]. Indeed, *GFAP*^*-/-*^*Vim*^*-/-*^ mice exhibit increased neurogenesis from endogenous NSPCs both under basal conditions and following hippocampal de-afferentation or perinatal hypoxia/ischemia [34, 36, 37]. Moreover, attenuation of reactive gliosis in *GFAP*^*-/-*^*Vim*^*-/-*^ mice leads to increased neuronal and astrocytic differentiation of adult rat hippocampal NSPCs transplanted into hippocampus [35] as well as improved integration and survival of retinal grafts [38]. Whether attenuation of reactive astrocytes affects the fate of human iPSC-derived NSPCs transplanted into stroke-injured brain is unknown.

Here we have assessed the effect of attenuation of reactive gliosis on the behavior of human iPSC-derived NSPCs at 8 weeks after transplantation into a model of cortical stroke using *GFAP*^*-/-*^*Vim*^*-/-*^ mice. We provide evidence that the reactive gliosis associated with cortical stroke in mice does not affect neurogenesis from intracortically grafted human-derived, reprogrammed NSPCs. However, due to the xenograft situation in the present study, it needs to be considered to what extent rodent models are suitable to reveal the impact of the ischemic environment on the grafted human cells.

## Materials and methods

### Animals and experimental design

We used adult male mice (10–12 weeks old, body weight 22–27 g) carrying a null mutation in the GFAP and vimentin genes (*GFAP*^*-/-*^*Vim*^*-/-*^ mice, labeled as KO in the figures) on a mixed C57Bl/6-129Ola-129Sv genetic background [21, 39] (n = 9) and age and sex-matched WT controls (n = 6) generated by our in-house breeding facility. Mice were subjected to distal middle cerebral artery occlusion (dMCAO), and transplanted with green fluorescent protein (GFP)-labeled human iPSC-NSPCs at 1 week and sacrificed at 9 weeks following the ischemic insult. All animals were kept in 12 h light/12 h dark cycles with *ad libitum* access to food and water. All procedures used in the present study were conducted in accordance with the European Union Directive (2010/63/EU) on the subject of animal rights, and were approved by the committees for the use of laboratory animals at Gothenburg and Lund Universities and the Swedish Board of Agriculture.

### Distal middle cerebral artery occlusion

Mice were anesthetized with isoflurane mixed with air (3.0% induction; 1.5% maintenance). Mice were anesthetized with a mixture of isoflurane (3.0% induction; 1.5% maintenance) and air. All mice received local injection of Marcain for pain relief (20 μl of 2.5 mg/ml stock solution, Astra Zeneca). During the surgical procedure and in the early recovery period (2 h), mice were placed on a heating pad at 37 °C. Permanent occlusion of the distal branch of the right middle cerebral artery (dMCAO) was performed as previously described [40]. Briefly, the distal portion of the right middle cerebral artery was exposed and occluded by cauterization. The artery was then cut off to be sure that there was no remaining blood flow to the corresponding cortical region. During the first week after dMCAO, animals were provided with high calorie gel diet (DietGel^™^ Boost, clear H_2_O) and injected subcutaneously with Ringer’s solution in case of dehydration.

### Transplantation

Human iPSCs were differentiated to neural stem precursor cells (NSPCs) via an embryoid body step as previously described [41]. Briefly, upon plating, embryoid bodies generated neural rosettes, which were carefully picked, dissociated and grown in adhesion as iPSC-NSPC line in the presence of 10 ng/ml FGF2, 10 ng/ml EGF (both from R&D systems) and B27 (1:1000, Invitrogen). The generated human iPSC-NSPC line was routinely cultured in monolayer on 0.1 mg/ml poly-L-ornithine- and 10 mg/ml laminin- (both from Sigma) coated plates and passaged at a ratio of 1:2 to 1:3 every second to third day using trypsin (Sigma).

Intracortical implantation of human iPSC-NSPCs, transduced with lentivirus carrying GFP and characterized in previous publications [5, 6, 41], was performed stereotaxically at 7 days after MCAO. On the day of surgery, human iPSC-NSPCs were resuspended to a final concentration of 100 000 cells/μl. A volume of 1 μl was injected at the following coordinates (from bregma and brain surface): anterior/posterior: +0.5 mm; medial/lateral: +1.8 mm; dorsal/ventral: -0.9 mm. Tooth-bar was set at -3.3 mm. Mice were injected subcutaneously with 10 mg/kg Cyclosporine A every day during the first month after transplantation and every other day during the second month.

### Immunohistochemistry

Mice were deeply anaesthetized with an overdose of pentobarbital and transcardially perfused with cold saline followed by 4% paraformaldehyde (PFA). Brains were post-fixed overnight in 4% PFA and incubated in 20% sucrose for 24 h at +4°C before being snap-frozen with dry ice, cut in 30 μm thick coronal sections on a microtome and stored in antifreeze solution at -20°C until they were used.

Sections were preincubated in blocking solution for 1 h (5% normal serum and 0.25% Triton X-100 in 0.1 M potassium-phosphate buffered solution). Primary antibodies were diluted in the blocking solution and incubated overnight at +4°C (S1 Table). Fluorophore-conjugated secondary antibodies (Molecular Probes or Jackson Laboratories) were diluted in blocking solution (1:200) and applied for 2 h at room temperature. Nuclei were stained with Hoechst (Molecular Probes or Jackson Laboratories) for 10 min and sections were mounted with Dabco mounting medium. Images were obtained using epifluorescence (Olympus, Germany) and confocal (Zeiss, Germany) microscopes.

Single labeling for NeuN was performed followed by biotin-avidin amplification. Briefly, after incubation with the primary rabbit anti-NeuN antibody, the samples were incubated with biotinylated secondary horse anti-rabbit antibody (1:200) and the staining visualized with avidin-biotin-peroxidase complex (Elite ABC kit, Vector Laboratories), followed by peroxidase-catalyzed diaminobenzidine (DAB) reaction.

### Quantifications and statistical analysis

All quantifications were performed by researchers blinded to the experimental groups. Lesion volume was assessed in NeuN-immunostained sections. Intact areas, identified by NeuN^+^ cells in the ipsilateral and contralateral hemispheres, were delineated and then measured using C.A.S.T. software (Visiopharm, Denmark). The lesion area was calculated by subtracting the non-lesioned (stained) area in the injured hemisphere from the corresponding area in the contralateral hemisphere. Lesion volume was then obtained by multiplying the lesion area by the thickness and distance between the sections (240 μm).

To evaluate the magnitude of glial reaction following stroke, we analyzed S100β immunoreactivity in a region of 1 mm around the stroke lesion or 300 μm around the graft. To evaluate the glial reaction around the stroke lesion, one picture per section was acquired at 4x magnification in the epifluorescence microscope, and a total of 3 sections per mouse were analyzed. The area of S100β-immunoreactivity was determined by image analysis using CellSens Dimension 2010 software (Olympus, Germany). In each section, areas of immunoreactivity were identified using a defined threshold for specific signal. Using these defined parameters, the images of each region were analyzed by the software, which calculated the total area covered by pixels/specific immunopositive signal. The values corresponding to total fluorescence areas were averaged and expressed as the percentage of area covered by S100β per animal. The same procedure was used to analyze the glial reaction around the graft.

To evaluate the activation of monocytes/microglia in response to the stroke, we counted ED1^+^ and Iba1^+^ cells in the area adjacent to the ischemic lesion in three coronal sections at +0.62, +0.86, and +1.1 mm from bregma using an epifluorescence microscope with 40x objective. A 1 mm region around the injury not containing the transplant was delineated and analyzed. To quantify ED1^+^ and Iba1^+^ cells in the region of the transplant, we analyzed a 300 μm area surrounding the graft in all sections containing the transplant. Total number of positive cells was estimated stereologically using C.A.S.T.-Grid software. Around 200 cells per animal were counted in a predefined fraction of the area of interest.

Total numbers of GFP^+^ cells in the grafts were estimated stereologically, selecting the whole graft as area of interest. GFP^+^ cells expressing DCX, NeuN, Ki67 and S100β were analyzed in the same way and results were expressed as percentage of the total number of GFP^+^ cells. Colocalization of different markers was in all cases validated with a confocal microscope (Zeiss, Germany).

Statistical comparisons were performed using GraphPad Prism version 7 (GraphPad software, La Jolla, CA, www.graphpad.com) by unpaired t-test. Data were presented as means ± SEM, and differences were considered significant at p < 0.05.

## Results

### Attenuation of reactive gliosis does not alter infarct volume after distal middle cerebral artery occlusion in mice

We first aimed to determine whether the extent of the ischemic lesion was affected in *GFAP*^*-/-*^*Vim*^*-/-*^ mice. Previous reports have indicated increased or unchanged infarct volume in these mice after middle cerebral artery transection [23] or cortical photothrombotic stroke [42], respectively. We used a model of cortical stroke in which a distal branch of the middle cerebral artery is occluded, resulting in permanent cortical ischemia without reperfusion. We observed 100% survival rate in both WT (9/9) and *GFAP*^*-/-*^*Vim*^*-/-*^ (6/6) groups.

**Fig 1:**
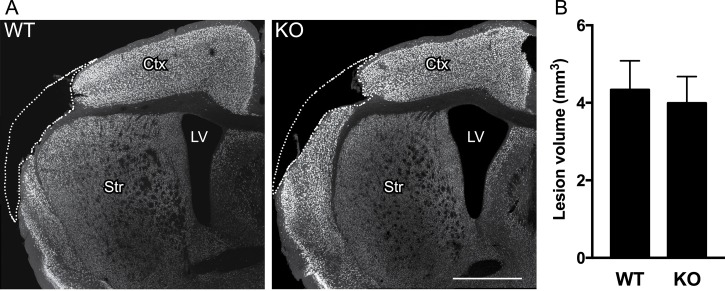
Genetic ablation of GFAP and vimentin does not alter volume of ischemic lesion following dMCAO. **(A)** Location of ischemic injury at 9 weeks after dMCAO, covering most of the somatosensory cortex, as shown by NeuN-immunostaining in coronal sections from WT and *GFAP*^*-/-*^*Vim*^*-/-*^ mice. Scale bar = 1 mm. **(B)** Mean volume of ischemic lesion in WT (n = 9) and *GFAP*^*-/-*^*Vim*^*-/-*^ (n = 6) mice. Means ± SEM. KO = *GFAP*^*-/-*^*Vim*^*-/-*^ mice.

At 7 days after dMCAO, WT and *GFAP*^*-/-*^*Vim*^*-/-*^ mice were transplanted with 100 000 GFP-labeled hiPSC-NSPCs in the cortex adjacent the stroke lesion (within 1 mm from the lesion border). All mice were sacrificed 8 weeks after transplantation. We analyzed in detail the distribution and extent of the ischemic injury in NeuN-stained sections to detect any possible alteration due to the attenuated reactive gliosis. At 9 weeks after stroke, neuronal loss was confined to the somatosensory cortex in both WT and *GFAP*^*-/-*^*Vim*^*-/-*^ mice with no differences in either location (Fig 1A) or volume of the ischemic damage (Fig 1B).

### Attenuation of reactive gliosis does not alter survival or neurogenic potential of human-iPSC-derived neural progenitors transplanted in stroke-injured mice

**Fig 2:**
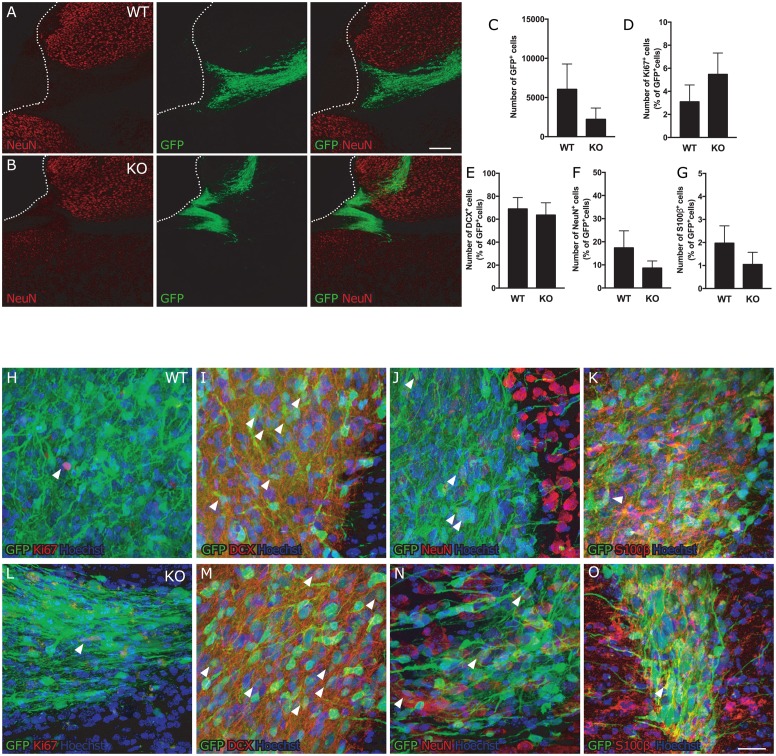
Attenuated reactive gliosis does not alter fate of human iPSC-NSPCs after transplantation in stroke-injured mice. **(A-B)** Distribution of GFP^+^ human iPSC-NSPCs in the vicinity of the cortical stroke lesion in WT and *GFAP*^*-/-*^*Vim*^*-/-*^ mice. Dotted line delineates the stroke lesion border, solid lines indicate the corpus callosum; corpus callosum = cc, cortex = ctx, striatum = str, transplant = tr. Scale bar = 200μm. **(C)** Number of GFP^+^ human iPSC-NSPCs remaining in the tissue 8 weeks after transplantation. Means ± SEM. **(D-G)** Percentage of transplanted cells which are actively proliferating (Ki67^+^, D) (WT n = 6; KO n = 4), giving rise to neural progenitor cells (DCX^+^, E) (WT n = 8; KO n = 4), neurons (NeuN^+^, F) (WT n = 7; KO n = 6) or glial cells (S100β^+^, G) (WT n = 9; KO n = 5) out of the total number of GFP^+^ human iPSC-NSPCs. **(H-O)** Representative images of transplanted GFP^+^ cells co-expressing the proliferation marker Ki67 (H, L), the neuroblast marker DCX (I, M), the mature neuronal marker NeuN (J, N) and the glial marker S100β (K, O) from a WT (H-K) and *GFAP*^*-/-*^*Vim*^*-/-*^ (I-O) mouse. Arrowheads indicate representative double positive cells. Scale bar = 20μm. KO = *GFAP*^*-/-*^*Vim*^*-/-*^ mice.

To evaluate the impact of the attenuated reactive gliosis on the fate of the human iPSC-NSPCs transplanted into stroke-injured mice, we first analyzed their survival and distribution. In both WT and *GFAP*^*-/-*^*Vim*^*-/-*^ mice, the human iPSC-NSPCs were located in the cortex up to 1 mm from the ischemic injury. We observed the same variability in the distributional pattern of grafted cells in WT and *GFAP*^*-/-*^*Vim*^*-/-*^ mice (Fig 2A and 2B). There was no significant difference between the two groups in the number of GFP^+^ grafted cells at 8 weeks after implantation (Fig 2C), but we observed a substantial variation in cell numbers in both WT and *GFAP*^*-/-*^*Vim*^*-/-*^ mice.

Next we assessed the effect of the attenuation of reactive gliosis on the proliferation of transplanted human iPSC-NSPCs. We found a lower percentage of Ki67^+^/GFP^+^ cells of the total number of GFP^+^ cells as compared to our previous reports [5, 43]. However, no differences were observed between WT and *GFAP*^*-/-*^*Vim*^*-/-*^ mice (Figs 2D, 2H, 2L and S1A). We then analyzed the phenotype of the transplanted cells in order to unveil a possible influence of the environment on their fate. Irrespective of the attenuation of the glial reaction, the ratio between neuroblasts (Figs 2E, 2I, 2M and S1B) (DCX^+^/GFP^+^) and mature neurons (Figs 2F, 2J, 2N and S1C) (NeuN^+^/GFP^+^) remained the same as previously observed [5, 43]. Moreover, the number of glial cells (Figs 2G, 2K, 2O and S1D) (S100β^+^/GFP^+^) did not differ between *GFAP*^*-/-*^*Vim*^*-/-*^ and WT mice and was almost 10 times lower than the number of mature neurons (Fig 2F and 2G): Taken together, these findings indicate that the attenuation of reactive gliosis does not alter the differentiation of human iPSC-NSPCs towards neurons or astrocytes.

### Attenuation of reactive gliosis does not alter inflammatory tissue environment after distal middle cerebral artery occlusion in mice

The inflammatory reaction after stroke involves many different cellular players, which interact with each other giving rise to the formation of a glial scar around the border of the ischemic lesion composed of reactive astrocytes, activated microglia, monocyte-derived macrophages (MDMs) and extracellular components [44]. Here we analyzed if the attenuation of reactive gliosis following stroke affects microglia and MDM activation and their recruitment to the site of injury.

**Fig 3:**
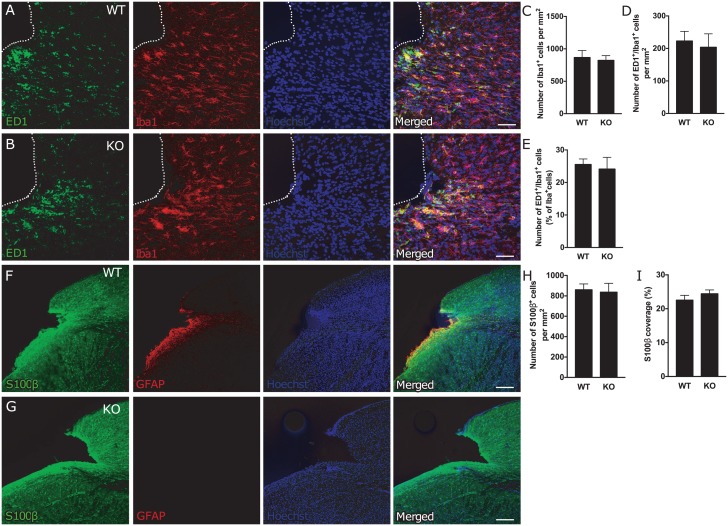
Attenuation of reactive gliosis does not alter number or activation of microglia/macrophages and the number of astrocytes in the region adjacent to the ischemic lesion. **(A-B)** Representative images of microglia/macrophage activation, as shown by ED1 (green) and Iba1 (red) immunostaining, around the stroke lesion in WT (A) and *GFAP*^*-/-*^*Vim*^*-/-*^ (B) mice. Scale bar = 50μm. **(C-E)** Total number of microglia/macrophages (Iba1^+^ cells, C), and number (ED1^+^/Iba1^+^ cells, D) and percentage (ED1^+^/Iba1^+^ cells of total number of Iba1^+^ cells, E) of activated microglia in the area around the ischemic lesion up to 1 mm from the lesion core in WT (n = 9) and *GFAP*^*-/-*^*Vim*^*-/-*^ (n = 6) mice. Means ± SEM. KO = *GFAP*^*-/-*^*Vim*^*-/-*^ mice. **(F-G)** Representative confocal images of the cortical lesion in WT and *GFAP*^*-/-*^*Vim*^*-/-*^ mice at 9 weeks after dMCAO, immunostained for GFAP (red) and S100β (green). Scale bar = 200 μm. **(H-I)** Quantification of astrocytes around the stroke lesion (1 mm around the lesion core) expressed as number of S100β^+^ cells per mm^2^ (H) and the relative S100β^+^ area (I) (WT n = 9; *GFAP*^*-/-*^*Vim*^*-/-*^ n = 6). Means ± SEM. KO = *GFAP*^*-/-*^*Vim*^*-/-*^ mice.

We observed an accumulation of cells immunoreactive for the microglia/macrophage marker Iba1 in the region adjacent to the ischemic lesion (Fig 3A and 3B), but their numbers did not differ between WT and *GFAP*^*-/-*^*Vim*^*-/-*^ mice (Fig 3C). Also the number of activated, ED1^+^/Iba1^+^ microglia/macrophages (Fig 3A, 3B and 3D) and percentage of ED1^+^/Iba1^+^ cells of the total number of Iba1^+^ cells (Fig 3E) were similar in the two groups.

Astrocytes around the ischemic lesion were visualized with antibodies against S100β, previously shown to be a reliable marker of mature astrocytes in both WT and *GFAP*^*-/-*^*Vim*^*-/-*^ mice [20]. Glial cells immunoreactive for S100β were distributed throughout cortex, with a higher density at the lesion border (Fig 3F and 3G). Similar numbers of these cells were detected around the stroke lesion in *GFAP*^*-/-*^*Vim*^*-/-*^ and WT mice (Fig 3H). In agreement, the area covered by astrocytes in the region adjacent to the injury calculated as percentage of the total area covered by S100β immunoreactivity did not differ between WT and *GFAP*^*-/-*^*Vim*^*-/-*^ mice (Fig 3I).

**Fig 4:**
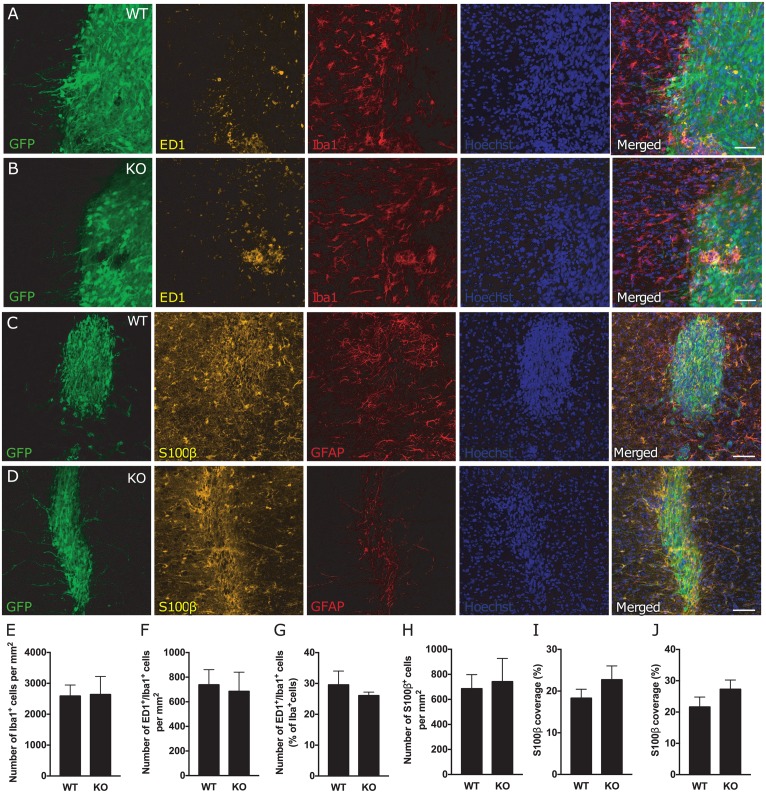
Attenuation of reactive gliosis does not alter numbers or activation of microglia/macrophages and the number of astrocytes in the region of the transplant after dMCAO. **(A-B)** Representative images of microglia/macrophage activation via ED1 (yellow) and Iba1 (red) staining, around the transplant (GFP^+^ cells, green) in WT (A) and KO (B) mice. Scale bar = 50μm. **(C-D)** Representative confocal images of a transplant in WT and *GFAP*^*-/-*^*Vim*^*-/-*^ mice at nine weeks after dMCAO showing the transplanted cells (GFP^+^ cells, green) and astrocytes stained for S100β (yellow) and GFAP (red). Scale bar = 100 μm. **(E-G)** Total number of microglia/macrophages (Iba1^+^ cells, E) and number (ED1^+^/Iba1^+^ cells, F) and percentage (ED1^+^/Iba1^+^ cells of total number of Iba1^+^ cells, G) activated microglia in the area inside and around the transplant in WT (n = 9) and *GFAP*^*-/-*^*Vim*^*-/-*^ (n = 4) mice. Means ± SEM. KO = *GFAP*^*-/-*^*Vim*^*-/-*^ mice. **(H-J)** Quantification of astrocytes in the area of the transplant (including the area 100 μm from the transplant) expressed as number of S100β^+^ cells per mm^2^ (H) and the relative S100β^+^ area around (I) and inside (J) the transplant (GFP^+^ cells, green) (WT n = 8; *GFAP*^*-/-*^*Vim*^*-/-*^ n = 5). Means ± SEM. KO = *GFAP*^*-/-*^*Vim*^*-/-*^ mice.

We finally assessed whether the inflammatory reaction in the region of the transplant was affected in *GFAP*^*-/-*^*Vim*^*-/-*^ mice. Similar to what was found around the stroke lesion, we observed a higher density of microglia/macrophages and glial cells inside and around the transplant. There was a comparable number of Iba1^+^ cells (Fig 4A, 4B and 4E), number of activated ED1^+^/Iba1^+^ microglia/macrophages (Fig 4F) and percentage of ED1^+^/Iba1^+^ cells of the total number of Iba1^+^ cells (Fig 4G) in WT and *GFAP*^*-/-*^*Vim*^*-/-*^ mice. Also, the number of S100β positive cells (Fig 4C, 4D and 4H) and the relative S100β positive area (Fig 4I and 4J) were comparable in WT and *GFAP*^*-/-*^*Vim*^*-/-*^ mice in the region of the graft.

## Discussion

The glial scar has been considered one of the barriers for CNS regeneration after injury, at least in some neurological disorders [11, 19, 26, 45–47]. Here we used a mouse model of genetically attenuated reactive gliosis (mice deficient for IF proteins GFAP and vimentin) to determine the impact of ischemia-induced reactive gliosis on the regenerative capacity of transplanted human iPSC-derived NSPCs. We find that attenuated reactive gliosis after cortical stroke does not influence neurogenesis or commitment towards glial lineage of intracortically grafted human iPSC-NSPCs at 8 weeks after transplantation. Moreover, in this model, attenuated reactive gliosis does not seem to alter the number and activation of microglia/macrophages and number of astrocytes around the stroke lesion and the graft.

In our study, the genetic ablation of GFAP and vimentin did not affect the size of the ischemic injury in the dMCAO model. This finding is in contrast to the notion that reactive astrocytes are important for neuroprotection in the ischemic penumbra, as evidenced by the decreased ability of *GFAP*^*-/-*^*Vim*^*-/-*^ astrocytes to counteract hypoosmotic stress [25], by the reduced ability of *GFAP*^*-/-*^*Vim*^*-/-*^ astrocytes to protect co-cultured neurons and to eliminate free oxygen species under oxygen and glucose deprivation and reperfusion [48], and by the lower survival of cells in the inner retina of *GFAP*^*-/-*^*Vim*^*-/-*^ mice in a retinal ischemia-reperfusion model [49]. After middle cerebral artery transection, the *GFAP*^*-/-*^*Vim*^*-/-*^ mice had a larger infarct volume compared to WT mice [23], whereas in a photothrombotic model of stroke [42] and here, after dMCAO, there were no differences between *GFAP*^*-/-*^*Vim*^*-/-*^ and WT mice in the size of the ischemic lesion. All these models lead to minimal reperfusion and photothrombotic stroke and dMCAO are characterized by restricted lesions, confined to the cerebral cortex. We previously reported an altered distribution of chondroitin sulphate proteoglicans (CSPGs), glial scar components inhibiting post-traumatic neuronal regeneration, in *GFAP*^*-/-*^*Vim*^*-/-*^ mice subjected to phototrombotic stroke, indicating decreased CSPG expression within the lesion boundary zone and increased expression within the lesion cortex [42]. Although we did not assess the distribution of CSPGs in the current study, it is possible that altered distribution of CSPGs around the infarct and the transplant in mice with attenuated reactive gliosis affected the survival and neuronal differentiation of the transplanted cells. Taken together, these findings seem to indicate that the extent of the ischemia-induced damage in *GFAP*^*-/-*^*Vim*^*-/-*^ mice is highly dependent on the specific stroke model used.

We observed that attenuated reactive gliosis did not affect neurogenesis or glial differentiation of intracortically grafted human iPSC-NSPCs after stroke. We previously showed that the *GFAP*^*-/-*^*Vim*^*-/-*^ mice exhibit increased basal and post-traumatic neurogenesis [34, 36] as well as increased neurogenesis after neonatal hypoxic-ischemic injury [37]. We also demonstrated better integration of neural grafts transplanted into retinas of *GFAP*^*-/-*^*Vim*^*-/-*^ mice [38] and increased neuronal differentiation of transplanted rat NSPCs in the hippocampus of *GFAP*^*-/-*^*Vim*^*-/-*^ mice [35]. However, these studies assessed neurogenesis and astrogenesis in the intact brain or in other injury models, whereas in the present study adult mice were subjected to ischemic cortical stroke in combination with a human-derived graft and immunosuppression. Astrocytes are known to respond differently to various types of brain damage, exhibiting protective or detrimental functions [14, 19, 24]. A transcriptome analysis of reactive astrocytes provides further support for the notion that phenotype of reactive astrocytes strongly depends on the type of injury [18, 24]. The analysis by Zamanian et al. [24], as well as our previous data, indicated that after transient MCAO followed by reperfusion, reactive astrocytes have predominantly a beneficial/protective role [23, 24]. The present findings provide evidence that the attenuation of astrocyte reactivity following permanent cortical ischemia does not sufficiently alter the environment to influence neurogenesis from the transplanted human-derived NSPCs in immunosuppressed mouse hosts.

The origin of the transplanted cells and the use of immunosuppression may be of particular importance with regard to outcome. Indeed, besides the presence of an ischemic lesion, important differences between the present experiment and the study showing increased neurogenesis and astrogenesis from grafted rat NSPCs into intact hippocampus of *GFAP*^*-/-*^*Vim*^*-/-*^ mice on the *Rag-1*^*-/-*^ background, i.e. lymphocyte-deficient [35], are the origin of the xenograft and immunosuppression by cyclosporine A versus Rag-1 deficiency. Human and rodent NSPCs exhibit different features such as growth requirements [50–52], differentiation kinetics [53], surface protein expression (i.e., aquaporins) [54] and susceptibility to different chemical compounds [55]. Therefore, it seems possible that the human-derived NSPCs respond differently to signals in the mouse tissue environment as compared to rat cells, not least because the NSPCs used here had been generated by reprogramming of human skin cells. It should also be mentioned that, although it was given to both *GFAP*^*-/-*^*Vim*^*-/-*^ and WT mice, cyclosporin A, which *per se* can have direct effects on neurogenesis [56], might have confounded the data interpretation.

The absence of effect of the attenuation of reactive gliosis on neuronal differentiation of human NSPCs could also be due to the fact that astrocytes are of mouse origin. Astrocytes are crucial for proper neuronal function [19, 57, 58] and exhibit many interspecies differences: astrocyte-to-neuron ratio is higher in humans than in rodents [59, 60], structure, morphology and variety of human astrocytes greatly differ from those of rodent astrocytes with human astrocytes exhibiting higher complexity than rodent ones [61]. Therefore, the interaction between transplanted NSPCs of human or rodent origin and murine reactive astrocytes, such as regulation of neuronal differentiation of NSPCs via Notch signaling [36, 62], could greatly differ.

We and others have suggested an interplay between microglia/macrophage activation and astrocyte reaction to injury [63–66]. We also recently demonstrated a tight relation between MDMs and reactive astrocytes, as the depletion of circulating monocytes early after stroke caused an overall reduction of astrocyte activation [43]. In line with previous studies, [23, 43] we observed here that the attenuation of reactive gliosis influenced neither microglia/macrophage reactivity nor number and coverage of S100β astrocytes around the stroke lesion and the graft. Interestingly, this is in contrast to increased presence and/or reactivity of microglia that we previously reported in *GFAP*^*-/-*^*Vim*^*-/-*^ mice that were crossed with the Batten disease mouse model [65].

In conclusion, our data suggest that post-stroke reactive gliosis in mice does not affect neuronal differentiation of intracortically grafted human iPSC-NSPCs. However, the implications of these findings for a clinical setting, in which such cells would be implanted into the stroke-injured human brain, need careful consideration due to the xenograft situation. Future studies to determine whether human reactive astrocytes affect neurogenesis from human NSPCs in experimental models are highly warranted.

## Supporting information

S1 Fig Colocalization of GFP^+^ cells with differentiation markers. Orthogonal reconstructions from confocal z-series of transplanted GFP^+^ cells co-expressing the proliferation marker Ki67 (A), the neuroblast marker DCX (B), the mature neuron marker NeuN (C) and the glial marker S100β (D). Representative images from WT mice. Arrowhead indicates double positive cell. Scale bar = 20μm.
(PDF)

S1 Table Primary antibodies used for immunohistochemistry. (DOCX)

## References

[1] Donnan GA, Fisher M, Macleod M, Davis SM. Stroke. Lancet. 2008;371(9624):1612–23. doi: 10.1016/S0140-6736(08)60694-7 . 18468545 10.1016/S0140-6736(08)60694-7

[2] George PM, Steinberg GK. Novel Stroke Therapeutics: Unraveling Stroke Pathophysiology and Its Impact on Clinical Treatments. Neuron. 2015;87(2):297–309. doi: 10.1016/j.neuron.2015.05.041 . 26182415 10.1016/j.neuron.2015.05.041 PMC4911814

[3] Kokaia Z, Tornero D, Lindvall O. Transplantation of reprogrammed neurons for improved recovery after stroke. Prog Brain Res. 2017;231:245–63. doi: 10.1016/bs.pbr.2016.11.013 . 28554399 10.1016/bs.pbr.2016.11.013

[4] Oki K, Tatarishvili J, Wood J, Koch P, Wattananit S, Mine Y, et al Human-induced pluripotent stem cells form functional neurons and improve recovery after grafting in stroke-damaged brain. Stem Cells. 2012;30(6):1120–33. doi: 10.1002/stem.1104 . 22495829 10.1002/stem.1104

[5] Tornero D, Wattananit S, Gronning Madsen M, Koch P, Wood J, Tatarishvili J, et al Human induced pluripotent stem cell-derived cortical neurons integrate in stroke-injured cortex and improve functional recovery. Brain. 2013;136(Pt 12):3561–77. doi: 10.1093/brain/awt278 . 24148272 10.1093/brain/awt278

[6] Tornero D, Tsupykov O, Granmo M, Rodriguez C, Gronning-Hansen M, Thelin J, et al Synaptic inputs from stroke-injured brain to grafted human stem cell-derived neurons activated by sensory stimuli. Brain. 2017;140(3):692–706. doi: 10.1093/brain/aww347 . 28115364 10.1093/brain/aww347

[7] Igarashi Y, Utsumi H, Chiba H, Yamada-Sasamori Y, Tobioka H, Kamimura Y, et al Glial cell line-derived neurotrophic factor induces barrier function of endothelial cells forming the blood-brain barrier. Biochem Biophys Res Commun. 1999;261(1):108–12. doi: 10.1006/bbrc.1999.0992 . 10405331 10.1006/bbrc.1999.0992

[8] Chen Y, Swanson RA. Astrocytes and brain injury. J Cereb Blood Flow Metab. 2003;23(2):137–49. doi: 10.1097/01.WCB.0000044631.80210.3C . 12571445 10.1097/01.WCB.0000044631.80210.3C

[9] Sofroniew MV, Vinters HV. Astrocytes: biology and pathology. Acta Neuropathol. 2010;119(1):7–35. doi: 10.1007/s00401-009-0619-8 . 20012068 10.1007/s00401-009-0619-8 PMC2799634

[10] Sofroniew MV. Astrocyte barriers to neurotoxic inflammation. Nat Rev Neurosci. 2015;16(5):249–63. doi: 10.1038/nrn3898 . 25891508 10.1038/nrn3898 PMC5253239

[11] Pekny M, Nilsson M. Astrocyte activation and reactive gliosis. Glia. 2005;50(4):427–34. doi: 10.1002/glia.20207 . 15846805 10.1002/glia.20207

[12] Burda JE, Sofroniew MV. Reactive gliosis and the multicellular response to CNS damage and disease. Neuron. 2014;81(2):229–48. doi: 10.1016/j.neuron.2013.12.034 . 24462092 10.1016/j.neuron.2013.12.034 PMC3984950

[13] Liu Z, Chopp M. Astrocytes, therapeutic targets for neuroprotection and neurorestoration in ischemic stroke. Prog Neurobiol. 2016;144:103–20. doi: 10.1016/j.pneurobio.2015.09.008 . 26455456 10.1016/j.pneurobio.2015.09.008 PMC4826643

[14] Pekny M, Pekna M. Astrocyte reactivity and reactive astrogliosis: costs and benefits. Physiol Rev. 2014;94(4):1077–98. doi: 10.1152/physrev.00041.2013 . 25287860 10.1152/physrev.00041.2013

[15] Middeldorp J, Hol EM. GFAP in health and disease. Prog Neurobiol. 2011;93(3):421–43. doi: 10.1016/j.pneurobio.2011.01.005 . 21219963 10.1016/j.pneurobio.2011.01.005

[16] Choudhury GR, Ding S. Reactive astrocytes and therapeutic potential in focal ischemic stroke. Neurobiol Dis. 2016;85:234–44. doi: 10.1016/j.nbd.2015.05.003 . 25982835 10.1016/j.nbd.2015.05.003 PMC4644522

[17] Wilhelmsson U, Bushong EA, Price DL, Smarr BL, Phung V, Terada M, et al Redefining the concept of reactive astrocytes as cells that remain within their unique domains upon reaction to injury. Proc Natl Acad Sci U S A. 2006;103(46):17513–8. doi: 10.1073/pnas.0602841103 . 17090684 10.1073/pnas.0602841103 PMC1859960

[18] Hol EM, Pekny M. Glial fibrillary acidic protein (GFAP) and the astrocyte intermediate filament system in diseases of the central nervous system. Curr Opin Cell Biol. 2015;32:121–30. doi: 10.1016/j.ceb.2015.02.004 . 25726916 10.1016/j.ceb.2015.02.004

[19] Pekny M, Pekna M, Messing A, Steinhauser C, Lee JM, Parpura V, et al Astrocytes: a central element in neurological diseases. Acta Neuropathol. 2016;131(3):323–45. doi: 10.1007/s00401-015-1513-1 . 26671410 10.1007/s00401-015-1513-1

[20] Wilhelmsson U, Li L, Pekna M, Berthold CH, Blom S, Eliasson C, et al Absence of glial fibrillary acidic protein and vimentin prevents hypertrophy of astrocytic processes and improves post-traumatic regeneration. J Neurosci. 2004;24(21):5016–21. doi: 10.1523/JNEUROSCI.0820-04.2004 . 15163694 10.1523/JNEUROSCI.0820-04.2004 PMC6729371

[21] Pekny M, Johansson CB, Eliasson C, Stakeberg J, Wallen A, Perlmann T, et al Abnormal reaction to central nervous system injury in mice lacking glial fibrillary acidic protein and vimentin. J Cell Biol. 1999;145(3):503–14. . 10225952 10.1083/jcb.145.3.503 PMC2185074

[22] Lu YB, Iandiev I, Hollborn M, Korber N, Ulbricht E, Hirrlinger PG, et al Reactive glial cells: increased stiffness correlates with increased intermediate filament expression. FASEB J. 2011;25(2):624–31. doi: 10.1096/fj.10-163790 . 20974670 10.1096/fj.10-163790

[23] Li L, Lundkvist A, Andersson D, Wilhelmsson U, Nagai N, Pardo AC, et al Protective role of reactive astrocytes in brain ischemia. J Cereb Blood Flow Metab. 2008;28(3):468–81. doi: 10.1038/sj.jcbfm.9600546 . 17726492 10.1038/sj.jcbfm.9600546

[24] Zamanian JL, Xu L, Foo LC, Nouri N, Zhou L, Giffard RG, et al Genomic analysis of reactive astrogliosis. J Neurosci. 2012;32(18):6391–410. doi: 10.1523/JNEUROSCI.6221-11.2012 . 22553043 10.1523/JNEUROSCI.6221-11.2012 PMC3480225

[25] Ding M, Eliasson C, Betsholtz C, Hamberger A, Pekny M. Altered taurine release following hypotonic stress in astrocytes from mice deficient for GFAP and vimentin. Brain Res Mol Brain Res. 1998;62(1):77–81. . 9795147 10.1016/s0169-328x(98)00240-x

[26] Sofroniew MV. Molecular dissection of reactive astrogliosis and glial scar formation. Trends Neurosci. 2009;32(12):638–47. doi: 10.1016/j.tins.2009.08.002 . 19782411 10.1016/j.tins.2009.08.002 PMC2787735

[27] Faulkner JR, Herrmann JE, Woo MJ, Tansey KE, Doan NB, Sofroniew MV. Reactive astrocytes protect tissue and preserve function after spinal cord injury. J Neurosci. 2004;24(9):2143–55. doi: 10.1523/JNEUROSCI.3547-03.2004 . 14999065 10.1523/JNEUROSCI.3547-03.2004 PMC6730429

[28] Myer DJ, Gurkoff GG, Lee SM, Hovda DA, Sofroniew MV. Essential protective roles of reactive astrocytes in traumatic brain injury. Brain. 2006;129(Pt 10):2761–72. doi: 10.1093/brain/awl165 . 16825202 10.1093/brain/awl165

[29] Verkhratsky A, Sofroniew MV, Messing A, deLanerolle NC, Rempe D, Rodriguez JJ, et al Neurological diseases as primary gliopathies: a reassessment of neurocentrism. ASN Neuro. 2012;4(3). doi: 10.1042/AN20120010 . 22339481 10.1042/AN20120010 PMC3320215

[30] Sofroniew MV. Reactive astrocytes in neural repair and protection. Neuroscientist. 2005;11(5):400–7. doi: 10.1177/1073858405278321 . 16151042 10.1177/1073858405278321

[31] Ridet JL, Malhotra SK, Privat A, Gage FH. Reactive astrocytes: cellular and molecular cues to biological function. Trends Neurosci. 1997;20(12):570–7. . 9416670 10.1016/s0166-2236(97)01139-9

[32] Silver J, Miller JH. Regeneration beyond the glial scar. Nat Rev Neurosci. 2004;5(2):146–56. doi: 10.1038/nrn1326 . 14735117 10.1038/nrn1326

[33] Vallieres L, Campbell IL, Gage FH, Sawchenko PE. Reduced hippocampal neurogenesis in adult transgenic mice with chronic astrocytic production of interleukin-6. J Neurosci. 2002;22(2):486–92. . 11784794 10.1523/JNEUROSCI.22-02-00486.2002 PMC6758670

[34] Larsson A, Wilhelmsson U, Pekna M, Pekny M. Increased cell proliferation and neurogenesis in the hippocampal dentate gyrus of old GFAP(-/-)Vim(-/-) mice. Neurochem Res. 2004;29(11):2069–73. . 15662841 10.1007/s11064-004-6880-2

[35] Widestrand A, Faijerson J, Wilhelmsson U, Smith PL, Li L, Sihlbom C, et al Increased neurogenesis and astrogenesis from neural progenitor cells grafted in the hippocampus of GFAP-/- Vim-/- mice. Stem Cells. 2007;25(10):2619–27. doi: 10.1634/stemcells.2007-0122 . 17628017 10.1634/stemcells.2007-0122

[36] Wilhelmsson U, Faiz M, de Pablo Y, Sjoqvist M, Andersson D, Widestrand A, et al Astrocytes negatively regulate neurogenesis through the Jagged1-mediated Notch pathway. Stem Cells. 2012;30(10):2320–9. doi: 10.1002/stem.1196 . 22887872 10.1002/stem.1196

[37] Jarlestedt K, Rousset CI, Faiz M, Wilhelmsson U, Stahlberg A, Sourkova H, et al Attenuation of reactive gliosis does not affect infarct volume in neonatal hypoxic-ischemic brain injury in mice. PLoS One. 2010;5(4):e10397 doi: 10.1371/journal.pone.0010397 . 20442854 10.1371/journal.pone.0010397 PMC2861004

[38] Kinouchi R, Takeda M, Yang L, Wilhelmsson U, Lundkvist A, Pekny M, et al Robust neural integration from retinal transplants in mice deficient in GFAP and vimentin. Nat Neurosci. 2003;6(8):863–8. doi: 10.1038/nn1088 . 12845328 10.1038/nn1088

[39] Eliasson C, Sahlgren C, Berthold CH, Stakeberg J, Celis JE, Betsholtz C, et al Intermediate filament protein partnership in astrocytes. J Biol Chem. 1999;274(34):23996–4006. . 10446168 10.1074/jbc.274.34.23996

[40] Perez-de Puig I, Miro F, Salas-Perdomo A, Bonfill-Teixidor E, Ferrer-Ferrer M, Marquez-Kisinousky L, et al IL-10 deficiency exacerbates the brain inflammatory response to permanent ischemia without preventing resolution of the lesion. J Cereb Blood Flow Metab. 2013;33(12):1955–66. doi: 10.1038/jcbfm.2013.155 . 24022622 10.1038/jcbfm.2013.155 PMC3851905

[41] Koch P, Opitz T, Steinbeck JA, Ladewig J, Brustle O. A rosette-type, self-renewing human ES cell-derived neural stem cell with potential for in vitro instruction and synaptic integration. Proc Natl Acad Sci U S A. 2009;106(9):3225–30. doi: 10.1073/pnas.0808387106 . 19218428 10.1073/pnas.0808387106 PMC2651316

[42] Liu Z, Li Y, Cui Y, Roberts C, Lu M, Wilhelmsson U, et al Beneficial effects of gfap/vimentin reactive astrocytes for axonal remodeling and motor behavioral recovery in mice after stroke. Glia. 2014;62(12):2022–33. doi: 10.1002/glia.22723 . 25043249 10.1002/glia.22723 PMC4307923

[43] Laterza C, Wattananit S, Uoshima N, Ge R, Pekny R, Tornero D, et al Monocyte depletion early after stroke promotes neurogenesis from endogenous neural stem cells in adult brain. Exp Neurol. 2017;297:129–37. doi: 10.1016/j.expneurol.2017.07.012 . 28746827 10.1016/j.expneurol.2017.07.012

[44] Gao Z, Zhu Q, Zhang Y, Zhao Y, Cai L, Shields CB, et al Reciprocal modulation between microglia and astrocyte in reactive gliosis following the CNS injury. Mol Neurobiol. 2013;48(3):690–701. doi: 10.1007/s12035-013-8460-4 . 23613214 10.1007/s12035-013-8460-4 PMC4079114

[45] Cho KS, Yang L, Lu B, Feng Ma H, Huang X, Pekny M, et al Re-establishing the regenerative potential of central nervous system axons in postnatal mice. J Cell Sci. 2005;118(Pt 5):863–72. doi: 10.1242/jcs.01658 . 15731004 10.1242/jcs.01658 PMC1351228

[46] Pekny M, Wilhelmsson U, Pekna M. The dual role of astrocyte activation and reactive gliosis. Neurosci Lett. 2014;565:30–8. doi: 10.1016/j.neulet.2013.12.071 . 24406153 10.1016/j.neulet.2013.12.071

[47] Miller DW, Cookson MR, Dickson DW. Glial cell inclusions and the pathogenesis of neurodegenerative diseases. Neuron Glia Biol. 2004;1(1):13–21. doi: 10.1017/s1740925x04000043 . 16614753 10.1017/s1740925x04000043 PMC1435946

[48] de Pablo Y, Nilsson M, Pekna M, Pekny M. Intermediate filaments are important for astrocyte response to oxidative stress induced by oxygen-glucose deprivation and reperfusion. Histochem Cell Biol. 2013;140(1):81–91. doi: 10.1007/s00418-013-1110-0 . 23756782 10.1007/s00418-013-1110-0

[49] Wunderlich KA, Tanimoto N, Grosche A, Zrenner E, Pekny M, Reichenbach A, et al Retinal functional alterations in mice lacking intermediate filament proteins glial fibrillary acidic protein and vimentin. FASEB J. 2015;29(12):4815–28. doi: 10.1096/fj.15-272963 . 26251181 10.1096/fj.15-272963

[50] Vescovi AL, Parati EA, Gritti A, Poulin P, Ferrario M, Wanke E, et al Isolation and cloning of multipotential stem cells from the embryonic human CNS and establishment of transplantable human neural stem cell lines by epigenetic stimulation. Exp Neurol. 1999;156(1):71–83. doi: 10.1006/exnr.1998.6998 . 10192778 10.1006/exnr.1998.6998

[51] Galli R, Pagano SF, Gritti A, Vescovi AL. Regulation of neuronal differentiation in human CNS stem cell progeny by leukemia inhibitory factor. Dev Neurosci. 2000;22(1–2):86–95. doi: 10.1159/000017430 . 10657701 10.1159/000017430

[52] Culbreth ME, Harrill JA, Freudenrich TM, Mundy WR, Shafer TJ. Comparison of chemical-induced changes in proliferation and apoptosis in human and mouse neuroprogenitor cells. Neurotoxicology. 2012;33(6):1499–510. doi: 10.1016/j.neuro.2012.05.012 . 22634143 10.1016/j.neuro.2012.05.012

[53] Ostenfeld T, Joly E, Tai YT, Peters A, Caldwell M, Jauniaux E, et al Regional specification of rodent and human neurospheres. Brain Res Dev Brain Res. 2002;134(1–2):43–55. . 11947936 10.1016/s0165-3806(01)00291-7

[54] Cavazzin C, Ferrari D, Facchetti F, Russignan A, Vescovi AL, La Porta CA, et al Unique expression and localization of aquaporin-4 and aquaporin-9 in murine and human neural stem cells and in their glial progeny. Glia. 2006;53(2):167–81. doi: 10.1002/glia.20256 . 16206164 10.1002/glia.20256

[55] Malik N, Efthymiou AG, Mather K, Chester N, Wang X, Nath A, et al Compounds with species and cell type specific toxicity identified in a 2000 compound drug screen of neural stem cells and rat mixed cortical neurons. Neurotoxicology. 2014;45:192–200. doi: 10.1016/j.neuro.2014.10.007 . 25454721 10.1016/j.neuro.2014.10.007 PMC4302729

[56] Chow A, Morshead CM. Cyclosporin A enhances neurogenesis in the dentate gyrus of the hippocampus. Stem Cell Res. 2016;16(1):79–87. doi: 10.1016/j.scr.2015.12.007 . 26720914 10.1016/j.scr.2015.12.007

[57] Araque A, Carmignoto G, Haydon PG, Oliet SH, Robitaille R, Volterra A. Gliotransmitters travel in time and space. Neuron. 2014;81(4):728–39. doi: 10.1016/j.neuron.2014.02.007 . 24559669 10.1016/j.neuron.2014.02.007 PMC4107238

[58] Dallerac G, Rouach N. Astrocytes as new targets to improve cognitive functions. Prog Neurobiol. 2016;144:48–67. doi: 10.1016/j.pneurobio.2016.01.003 . 26969413 10.1016/j.pneurobio.2016.01.003

[59] Bass NH, Hess HH, Pope A, Thalheimer C. Quantitative cytoarchitectonic distribution of neurons, glia, and DNa in rat cerebral cortex. J Comp Neurol. 1971;143(4):481–90. doi: 10.1002/cne.901430405 . 4945394 10.1002/cne.901430405

[60] Leuba G, Garey LJ. Comparison of neuronal and glial numerical density in primary and secondary visual cortex of man. Exp Brain Res. 1989;77(1):31–8. . 2792267 10.1007/BF00250564

[61] Oberheim NA, Takano T, Han X, He W, Lin JH, Wang F, et al Uniquely hominid features of adult human astrocytes. J Neurosci. 2009;29(10):3276–87. doi: 10.1523/JNEUROSCI.4707-08.2009 . 19279265 10.1523/JNEUROSCI.4707-08.2009 PMC2819812

[62] Lebkuechner I, Wilhelmsson U, Mollerstrom E, Pekna M, Pekny M. Heterogeneity of Notch signaling in astrocytes and the effects of GFAP and vimentin deficiency. J Neurochem. 2015;135(2):234–48. doi: 10.1111/jnc.13213 . 26118771 10.1111/jnc.13213

[63] Gliem M, Krammes K, Liaw L, van Rooijen N, Hartung HP, Jander S. Macrophage-derived osteopontin induces reactive astrocyte polarization and promotes re-establishment of the blood brain barrier after ischemic stroke. Glia. 2015;63(12):2198–207. doi: 10.1002/glia.22885 . 26148976 10.1002/glia.22885

[64] Liddelow SA, Guttenplan KA, Clarke LE, Bennett FC, Bohlen CJ, Schirmer L, et al Neurotoxic reactive astrocytes are induced by activated microglia. Nature. 2017;541(7638):481–7. doi: 10.1038/nature21029 . 28099414 10.1038/nature21029 PMC5404890

[65] Macauley SL, Pekny M, Sands MS. The role of attenuated astrocyte activation in infantile neuronal ceroid lipofuscinosis. J Neurosci. 2011;31(43):15575–85. doi: 10.1523/JNEUROSCI.3579-11.2011 . 22031903 10.1523/JNEUROSCI.3579-11.2011 PMC3218425

[66] Kraft AW, Hu X, Yoon H, Yan P, Xiao Q, Wang Y, et al Attenuating astrocyte activation accelerates plaque pathogenesis in APP/PS1 mice. FASEB J. 2013;27(1):187–98. doi: 10.1096/fj.12-208660 . 23038755 10.1096/fj.12-208660 PMC3528309

