# Supplementary figures and images for: Attenuation of reactive gliosis in stroke-injured mouse brain does not affect neurogenesis from grafted human iPSC-derived neural progenitors
Source: PLoS One. 2018 Feb 5;13(2):e0192118. doi: 10.1371/journal.pone.0192118 (PMC5798785; doi:10.1371/journal.pone.0192118)

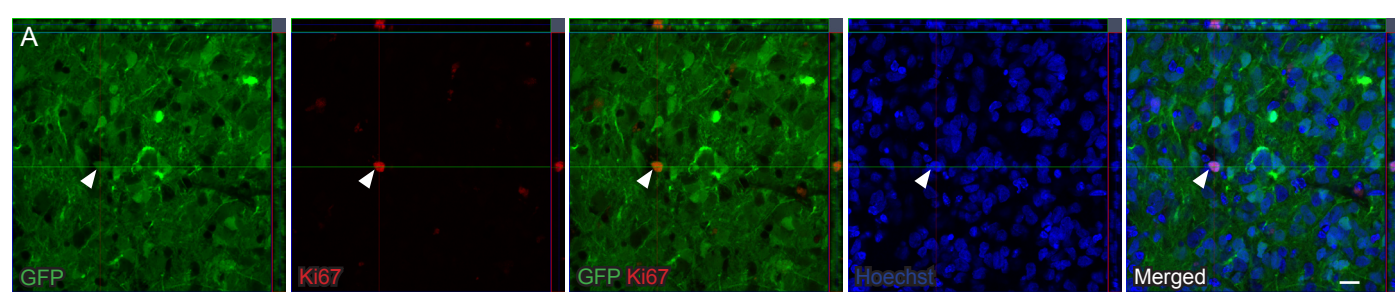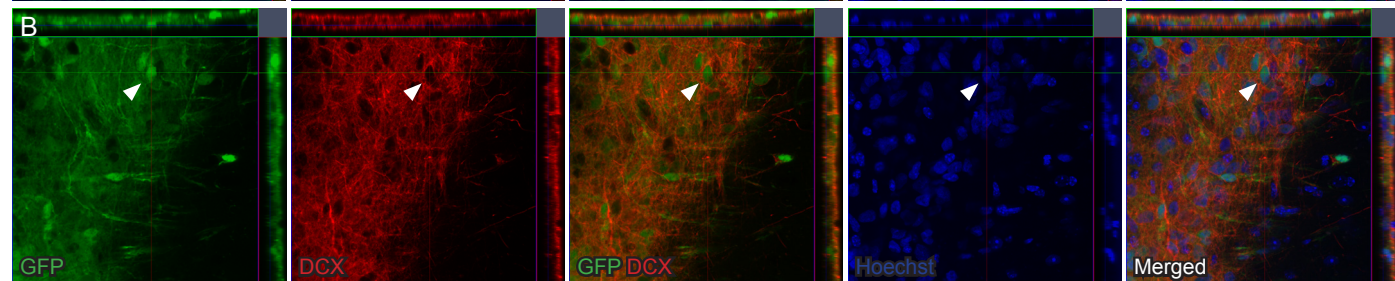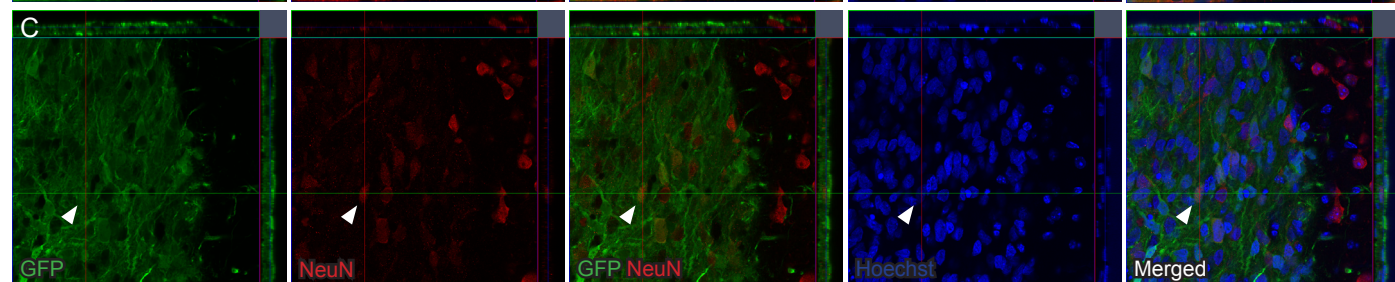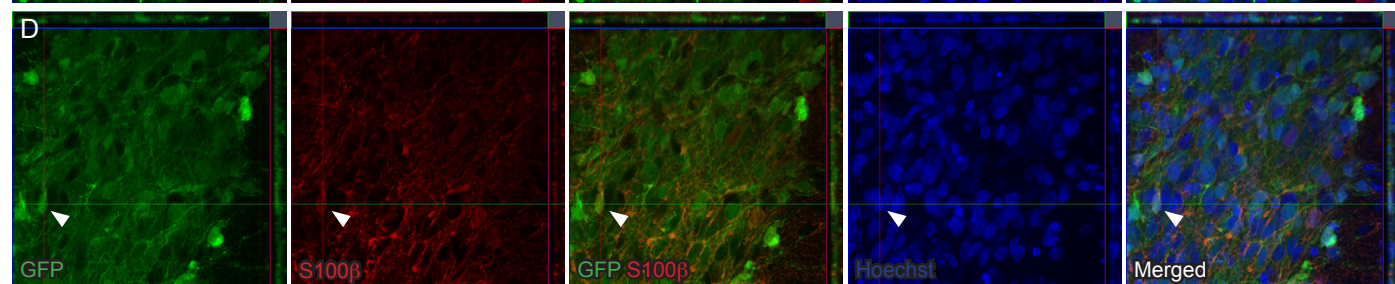

Supplement: S1 Fig — Orthogonal reconstructions from confocal z-series of transplanted GFP+ cells co-expressing the proliferation marker Ki67 (A), the neuroblast marker DCX (B), the mature neuron marker NeuN (C) and the glial marker S100β (D). Representative images from WT mice. Arrowhead indicates double positive cell. Scale bar = 20μm. (PDF) [file pone.0192118.s001.pdf]
