# Supplementary material for: Attenuation of reactive gliosis in stroke-injured mouse brain does not affect neurogenesis from grafted human iPSC-derived neural progenitors
Source: PLoS One. 2018 Feb 5;13(2):e0192118. doi: 10.1371/journal.pone.0192118 (PMC5798785; doi:10.1371/journal.pone.0192118)
Supplement: S1 Table — (DOCX) [file pone.0192118.s002.docx]

**S1 Table.** Primary antibodies used for immunohistochemistry

| **Antibodies** | **Host species** | **Dilution** | **Company** |
| --- | --- | --- | --- |
| CD68 (ED1) | Rat | 1:200 | AbD Serotec |
| DCX | Goat | 1:400 | Santa Crutz |
| GFAP | Rabbit | 1:400 | Zymed (ThemroFisher) |
| GFAP | Chicken | 1:1000 | Merk Millipore |
| GFP | Chicken | 1:3000 | Merk Millipore |
| Iba1 | Goat | 1:200 | AbD Serotec |
| Iba1 | Rabbit | 1:1000 | Wako |
| Ki67 | Rabbit | 1:250 | Abcam |
| NeuN | Rabbit | 1:2000 | Abcam |
| S100β | Rabbit | 1:400 | DAKO |
